# Feasibility of Round Window Stimulation by a Novel Electromagnetic Microactuator

**DOI:** 10.1155/2017/6369247

**Published:** 2017-10-29

**Authors:** Wouter Johannes van Drunen, Mathias Mueller, Anatoly Glukhovskoy, Rolf Salcher, Marc Christopher Wurz, Thomas Lenarz, Hannes Maier

**Affiliations:** ^1^Cluster of Excellence EXC 1077/1 “Hearing4all”, Department of Otolaryngology, Hannover Medical School, Carl-Neuberg-Str. 1, 30625 Hannover, Germany; ^2^Institute for Micro Production Technology, Leibniz Universität Hannover, An der Universität 2, 30823 Garbsen, Germany

## Abstract

**Introduction:**

Most implantable hearing aids currently available were developed to compensate the sensorineural hearing loss by driving middle ear structures (e.g., the ossicles). These devices are successfully used in round window (RW) stimulation clinically, although this was initially not the intended use. Here, a novel microactuator, specifically designed for RW stimulation, was tested in human temporal bones to determine actuator performance and applicability.

**Methods:**

Stapes footplate response to RW stimulation was determined experimentally in human temporal bones and the obtained sound pressure output level was estimated.

**Results:**

The actuator had a flat displacement response between 0.125 and 4 kHz, a resonance between 4 and 7 kHz, and a roll-off above. At increasing contact force, the stapes footplate displacement decreased by 5–10 dB re *μ*m for forces ≥ 2 mN. The equivalent sound pressure level between 0.125 and 4 kHz amounted to 87–97 eq dB SPL and increased to 117 eq dB SPL for frequencies of 4–7 kHz. The total harmonic distortion (THD) of the actuator ranged within 15–40% for static forces of 5 mN.

**Conclusion:**

The feasibility of an electromagnetic actuator that may be placed into the RW niche was demonstrated but requires further optimization in terms of THD and static force sensitivity.

## 1. Introduction

Most implantable hearing aids available on the market were developed for compensating sensorineural hearing losses by driving the middle ear structures (e.g., the ossicles) [1]. Only a single device was intended from the beginning to treat mixed hearing loss by circumventing the middle ear driving directly the perilymph of the inner ear by a piston [2]. Though most hearing aids were not designed to stimulate the cochlea via the round window membrane (RWM), they bear the potential to help those patients where the middle ear is absent or dysfunctional. Although Spindel et al. demonstrated already in 1995 successfully the stimulation of the RWM using an implantable device [1], it took a decade before this approach was clinically applied to patients [3–5]. Since then, the round window (RW) stimulation has developed to a successful treatment of mixed hearing losses [6, 7]. Due to the still high variability in the outcomes, research focusses on the investigation and optimization of the coupling between the implantable device and the RWM [8, 9]. Though the implantable hearing devices on the market perform well, most were originally not designed to stimulate the RW directly. In this study, we present a novel microactuator that combines precision engineering with microsystem technologies. This actuator, specially designed to fit into the round window niche, differs in size and working principle from other actuators [10] and was tested in human temporal bones to demonstrate feasibility and to determine the influence of controlled axial static force on actuator performance and sound transmission. The stapes footplate response to RW stimulation was determined experimentally in fresh human temporal bones and its sound pressure output level was estimated. Experiments were performed in analogy to the “ASTM standard (F2504-05) method for the output determination of implantable middle ear hearing devices (IMEHDs) in human cadaver temporal bones (TBs).”

## 2. Materials and Methods

### 2.1. Design of the Microactuator

**Figure 1:**
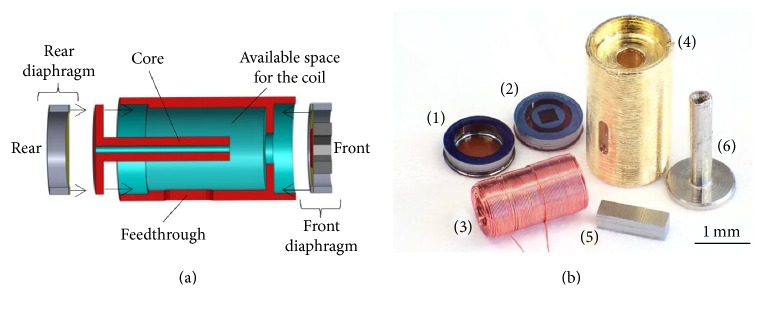
(a) Schematic drawing of the microactuator and (b) picture of the unassembled components (from left to right: rear (1) and front (2) diaphragm, coil (3), stator housing (4), coupling element without the spherical tip (5), and ferromagnetic NiFe45/55 core (6)).

A schematic drawing of the microactuator [11] is depicted in Figure 1(a). The microactuator consists of six components (Figure 1(b)). Five of the six components (stator housing, ferromagnetic core, coil, and coupling element) were manufactured with high precision by traditional processes. To prevent corrosion and for robustness, the housing, measuring 2.8 × 1.5 mm, was coated with a gold layer (*d* = approx. 200 nm).

**Figure 2:**
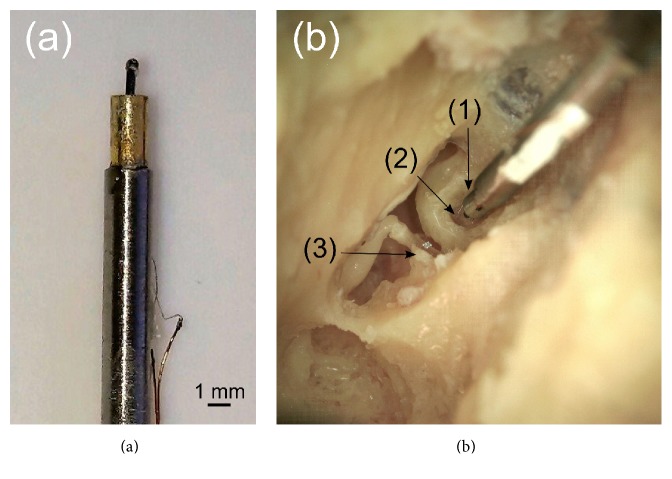
(a) Actuator glued on the holder. (b) Coupling of the actuator (1) to the RWM (2) and the stapes (3) used for measurements.

The front and the rear diaphragms were manufactured by microsystem technologies processes. The rear diaphragm, whose function is to compensate the pressure differences during operation of the actuator, consisted of a single polymer membrane, while the front diaphragm was a polymer membrane with a ferromagnetic backside (NiFe45/55). The same material was used for the ferromagnetic core. The front membrane had a superstructure to hold the coupling element intended for round window stimulation. The coupling element, mounted on the front membrane superstructure, consisted of a cuboid rod of *l* = 1.2 mm length on whose top an additional ceramic spherical tip (*d* = 500 *μ*m, Figure 2(a)) [12] was mounted. The spherical tip served as contact to the round window membrane (RWM). The feed through hole for the electrical contact wire was hermetically sealed using glue.

By driving the coil of the assembled microactuator with a current, a magnetic field is generated which attracts the ferromagnetic backside of the front diaphragm with the coupling element. Although these ferromagnetic materials are easier to handle, they bear the disadvantage of allowing only attractive force; consequently a bias voltage for bidirectional operation is required. Numerical model optimization studies showed that a 10 *μ*m air gap between the diaphragm magnetic back and coil core resulted in the best performance of the actuator.

Three of four actuators evaluated in this study had a coil with 1000 windings of a copper wire (*d* = 22 *μ*m) and are termed 09–22, 17–22, and 18–22. The fourth actuator had a wound coil of approx. 500 windings with a 30 *μ*m diameter copper wire (06–30).

### 2.2. Preparation of the Human Cadaver Temporal Bone

The human cadaver temporal bones (TB) used in this study (*n* = 2) were harvested within 48 hours postmortem and stored at −21°C for later use. The experiments were performed within a 12-hour time frame after thawing of the temporal bones at room temperature and preparations were periodically rinsed with saline during the experiments. By an extended posterior tympanotomy accompanied by a mastoidectomy, access to the middle ear cavity was obtained. The facial nerve was dissected and the round window niche was drilled carefully away, leaving approx. 0.2 to 0.5 mm of the surrounding bone intact. In experiments the temporal bone was mounted in a laboratory clamp on a vibration isolated table (LW3048B, Newport, Germany).

An acoustic stimulus to the TB was provided by a Beyer Dynamics DT-48 loudspeaker driven by a buffer amplifier (SA1, Tucker Davis Technologies) and connected to a sound applicator by a flexible tube. The sound applicator was cemented into the external ear canal using dental cement (Paladur, Heraeus Kulzer, Germany). A probe microphone (ER-7C, Etymotics, USA) was placed inside the sound applicator and the tip was positioned 1-2 mm away from the tympanic membrane. Loudspeaker and microactuator were driven sequentially by a custom written multisine signal with equal amplitudes (approx. 60 mV_RMS_ for the loudspeaker, 0.18 V_RMS_ per frequency for the actuators 09–22, 17–22, and 18–22, and 0.13 V_RMS_ per frequency for the actuator 06–30) at 0.125, 0.25, 0.5, 1, 2, 3, 4, 6, 8, and 10 kHz. For the actuators 09–22, 17–22, and 18–22, all inputs had an additional DC offset of 1.5 V whereas for the actuator 06–30 all inputs had an additional DC offset of 1.2 V. In the total harmonic distortion measurements the microactuator was driven by single sines waves at 0.25, 0.5, 1, and 2 kHz (approx. 1.06 V_RMS_ with the same DC offset of 1.5 V for the actuators 09–22, 17–22, and 18–22 and approx. 0.85 V_RMS_ with the same DC offset of 1.2 V for actuator 06–30). Additionally pseudorandom white noise signals (input = 18 mV_RMS_ for actuators 09–22, 17–22, and 18–22 and 13 mV_RMS_ for actuator 06–30) were applied to the actuator. Signals were generated by a data acquisition system VibSoft 4.8 (Polytec, Germany) at 25.6 kHz sample rate and a frequency resolution of 12.5 Hz (800 FFT lines). Measured response data were averaged 150 times. In single and multisine stimulation, the signal-to-noise-ratio (SNR) of acquired signals was computed by averaging three FFT lines below and above each frequency component as a noise floor estimate. Only data with a SNR ≥ 6 dB was included in the analysis. In the total harmonic distortion (THD) calculation only higher harmonics with SNR ≥ 12 dB and at frequencies ≤ 10 kHz were included. Pseudorandom white noise signals were smoothed with a moving average with a centered window size of 3 FFT lines.

Displacement amplitudes were measured by a Laser-Doppler Velocimeter (LDV) with a micromanipulator (OFV-5000, HLV-MM30, Polytec, Germany) mounted on a surgical microscope (OPMI-1, Zeiss, Germany). Small pieces of reflective tape (approx. 0.2 × 0.2 mm) were positioned on the stapes footplate and on the backside of the coupling element spherical tip to enhance the reflected signal intensity. For each measurement sequence, the incidence angle between the coupling element long axis or the stapes footplate normal and the LDV beam was estimated and a cosine correction was performed.

Prior to the experiments, the stapes footplate responses to acoustic stimulation at the tympanic membrane (~94 dB SPL) were measured and compared to the modified ASTM F2504-05 range [13] introduced by Rosowski et al. [14]. Only TBs compliant with the extended ASTM range were included in the analysis.

### 2.3. Coupling of the Actuator to the Round Window Membrane

The actuator was glued with its back side to the tip of a stainless steel rod (Figure 2(a)). The rod was mounted to a force sensor (LSB 210, Futek Advanced Sensor Technology, USA) attached to a 3-axis micromanipulator (MM3301, WPI, Germany) located on the vibration isolated table. Mounting the temporal bone and the actuator on separate magnetic holders allowed them to be oriented independently to each other (Figure 2(b)).

The actuator was positioned with its long axis visually perpendicular to the RWM surface. The actuator's spherical tip was placed close (*d* = ~100 *μ*m) to the RWM without physical contact to the membrane or bony surroundings. This position was taken as the starting point and the force sensor was zeroed. After defining the initial position, the actuator was displaced towards the RWM first in 50 *μ*m steps and from 0.2 mN on in 20 *μ*m steps. At each applied distance step (1) the SFP displacement in response to actuator stimulation and (2) in response to acoustic stimulation of the tympanic membrane as well as (3) the displacement of the actuator spherical tip was measured sequentially by LDV. The static RW load was documented right after applying a distance step and after the measurement sequence was finished (~10 min) assuming relaxation of the RWM into steady state after that elapsed time. The actuator was moved against the RWM until a static force range of ~0–5 mN was covered.

After the completion of the measurement sequence, the actuator was moved back to its initial position where no physical contact to the membrane or bony surroundings was observed and the actuator tip displacement was measured again to check for the actuators integrity.

### 2.4. Data Analysis

Results were analyzed using Matlab (MathWorks Inc., USA). The individual experimental results were compared to each other at selected static forces of 1, 2, 3, and 5 mN. LDV vibratory responses at these static forces were obtained by linear interpolation between adjacent experimental steps.

## 3. Results

### 3.1. Stapes Footplate Response to Acoustic Stimulation

Both human temporal bones fulfilled the modified ASTM criteria and were included in the analysis (see Figure 8 in the Supplementary Material available online at https://doi.org/10.1155/2017/6369247).

### 3.2. Loading Effect on the Actuator Displacement Output

**Figure 3:**
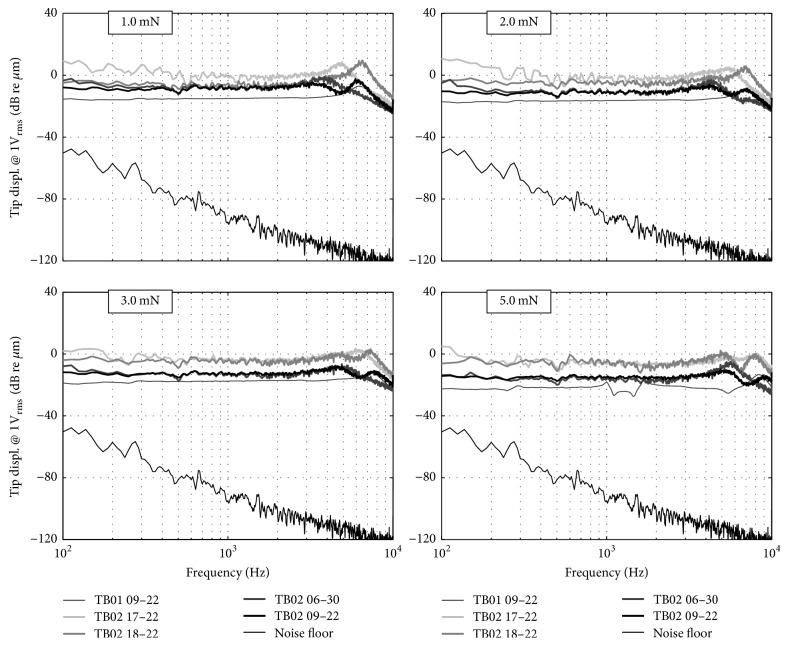
Tip displacement amplitudes of the actuators at different static RW loads normalized to 1 V_RMS_ actuator input.

Figure 3 depicts the measured tip displacement for pseudorandom white noise input signals at the actuator. All measurements results were outside the noise floor. At forces ≤ 5 mN, the measured tip displacement amplitudes had a plateau below the axial resonance of the actuators at 4–7 kHz. Displacement amplitudes at higher frequencies showed a roll-off (Figure 3). In TB02 all actuators show a slight resonance at 0.5 kHz whereas actuator 09–22 in TB01 shows a resonance at 1-2 kHz at a loading force of 5 mN.

### 3.3. Stapes Footplate Response to Round Window Stimulation

**Figure 4:**
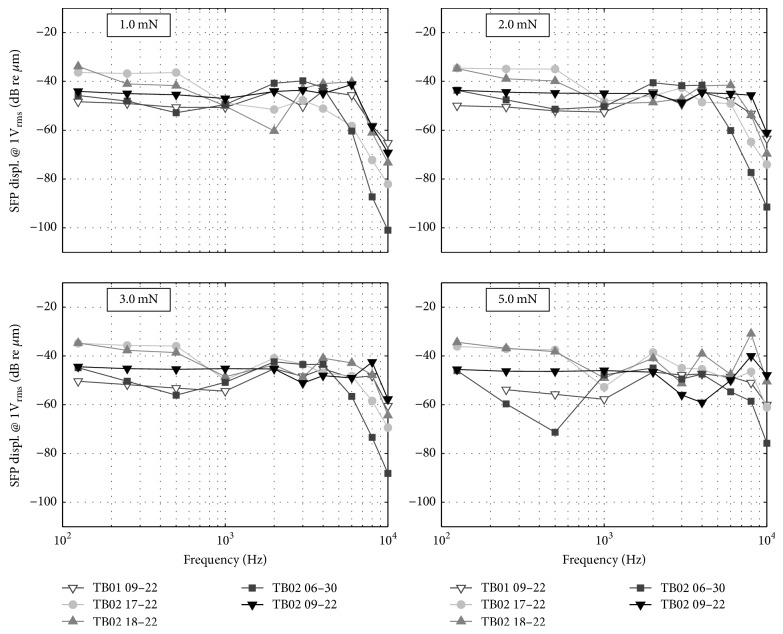
Stapes footplate displacement response to RW stimulation at different static loads normalized to 1 V_RMS_ input.

**Figure 5:**
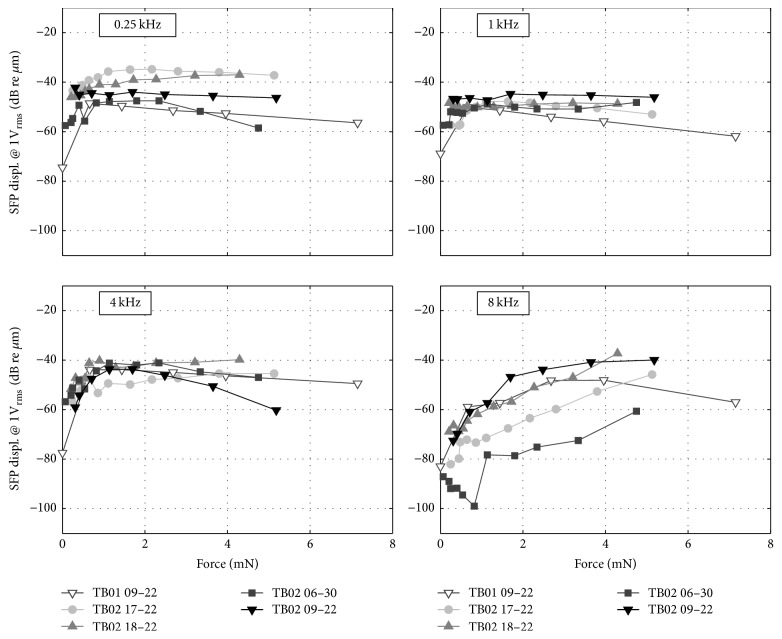
Stapes footplate displacement response to RW stimulation normalized to 1 V_RMS_ actuator input for different frequencies as a function of static load.

Figure 4 depicts the stapes footplate frequency responses at four different static coupling loads.  Figure 5 shows the same experimental data for four selected frequencies as a function of applied static force. Between 0.125 and 4 kHz SFP responses were flat at approx. −43 dB re *μ*m across all tested actuators (Figure 4). At frequencies > 4 kHz the SFP responses declined by approx. 35 dB/oct. At increasing force load (≥2 mN), the SFP displacement decreases slightly (also seen in Figure 5) and the roll-off in SFP displacement moved to higher frequencies. At the same time, a larger variance in the SFP displacement at frequencies below 6 kHz was observed.

### 3.4. Equivalent Sound Pressure Level Output

**Figure 6:**
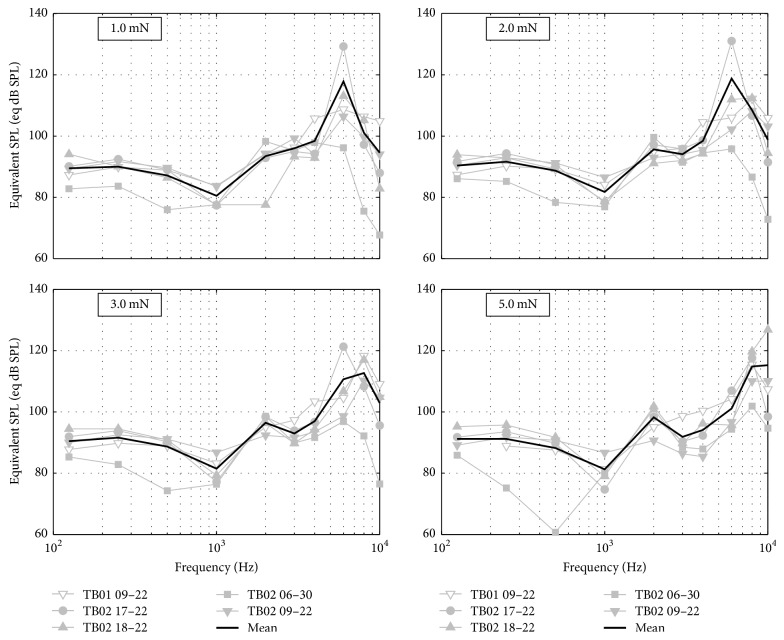
Equivalent sound pressure levels for different static round window membrane loads. The thick black line depicts the average equivalent sound pressure level.

From the SFP response to sound (Figure 8 in the online Supplementary Material) and to actuator stimulation for the different coupling forces, an estimate of the equivalent sound pressure level for a nominal input of 1 V to the actuator was calculated. The corresponding equivalent sound pressure level estimates for the four different RW loads are depicted in Figure 6. For loads ≤ 3 mN the equivalent sound pressure level increases from 87 dB at 0.125 kHz to around 97 dB at 4 kHz with a local minimum at 1 kHz. At a load of 5 mN, actuator 06–30 showed a clear reduction in the equivalent sound pressure level at 0.5 kHz.

### 3.5. Total Harmonic Distortion (THD)

**Figure 7:**
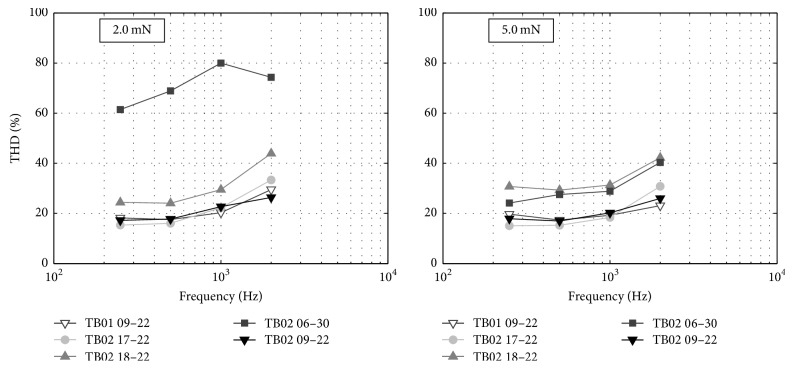
The total harmonic distortion of the actuators measured at the actuator tip for two different static RW loads.

Figure 7 depicts the THD of the actuator tip at different static RW loads. Highest harmonic distortions were found with actuator 06–30 at a load of 2 mN and were 60–80% but declined to 25–40% with increasing RW loads (5 mN). Actuators 17–22 and 09–22 showed THDs of 15–35% at static forces ≥ 2 mN that were mostly independent on increased RW load. THDs of actuator 18–22 highest loads (5 mN) were comparable to actuator 06–30 and were 20–40%.

## 4. Discussion

The frequency specific amount of stapes footplate displacement depends generally on the actuator performance and the coupling force between the actuator and the RWM [6] as well as the contact area between the coupling element and the RWM. Both the actuator tip displacement and the stapes footplate response to RW stimulation were measured for different contact forces and showed a flat displacement response of both the actuator tip and the stapes footplate between 0.125 and 4 kHz. At frequencies > 4 kHz the stapes footplate responses declined by approx. 35 dB/oct whereas the actuators showed a resonance in tip displacement at 4–8 kHz followed by a roll-off. At increasing force, the stapes footplate response decreases by 5–10 dB re *μ*m for static forces ≥ 2 mN, which is attributed to decrease in actuator tip displacement (data not shown).

As Figure 5 shows, maximal SFP responses to actuator stimulation were reached at static forces < 2 mN for frequencies ≤ 1 kHz and at forces of 2–4 mN for higher frequencies. We assume that the decrease of SFP vibration amplitude at higher forces is more likely due to actuator limitations than to a decline in transmission to the SFP. Saturation effects can be explained by the limited available space (~10 *μ*m) between the actuator front diaphragm and coil core that can potentially impair membrane movement when decreased at higher static loads.

The equivalent sound pressure level estimate between 0.125 and 4 kHz amounted to 87–97 dB and increased to 117 dB for frequencies > 4–7 kHz. With the exception of actuator 06–30, which showed a clear reduction in the equivalent sound pressure level at 0.5 kHz, the equivalent sound pressure level showed a flat response between 0.125 and 4 kHz. The decrease in equivalent sound pressure level of actuator 06–30 at 0.5 kHz may be attributed to variations in the actuator construction since actuator 06–30 was a precursor to actuators 09–22, 17–22, and 18–22. The total harmonic distortion of the actuators, measured at the actuator tip, ranged within 15–40% for static forces of 5 mN.

The presented novel microactuators were tested in human temporal bones to determine the feasibility of RW stimulation with an electromagnetic actuator of similar diameter as the RW and length to fit into the RW niche. Compared to the available systems in the market [7, 9], the actuator showed a good performance. Concerning speech relevant frequencies of 0.5–4 kHz the achieved SPLs were within 85–105 dB HL [15]. Assuming a minimum coverage of 30 dB dynamic range for sufficient speech intelligibility [16] the treatment of a potential hearing loss of 60 dB HL at low frequencies and more at higher frequencies appears to be feasible. The current observed maximum static force range of ≤10 mN for these actuators needs to be increased to allow handling and manual positioning of the device during the implantation procedure. The current actuator design uses ferromagnetic materials. Though these materials are easier to handle, their ferromagnetic behavior, when exposed to an external magnetic field, has the disadvantage that they only allow attractive forces. As a result, a DC offset was required to allow the coupling element move in both directions, making this design unattractive in terms of power requirement. One way to circumvent this bias voltage is the use of permanent magnetic materials. To reduce the THD of the actuator, further optimization of the diaphragm properties is required. To summarize, these novel microactuators show very promising results though further improvements are required for this actuator to be successful.

## 5. Conclusion

We could demonstrate the feasibility of the electromagnetic microactuator for RW stimulation with the appropriate dimensions to fit entirely into the RW niche. Although the output provided is sufficient to treat moderate to severe hearing loss, further optimization is required in terms of distortion and power consumption for hearing applications.

## Supplementary Material

Stapes footplate displacement response to acoustic stimulation of the tympanic membrane (~ 94 dB SPL). The mean responses (ASTM F2504-5) are presented as black line and the 95% percentile range is shown as grey area. The 20% wider range from Rosowski et al. [14] is shown as light grey area.
