# Supplementary figures and images for: Feasibility of Round Window Stimulation by a Novel Electromagnetic Microactuator
Source: Biomed Res Int. 2017 Oct 29;2017:6369247. doi: 10.1155/2017/6369247 (PMC5682051; doi:10.1155/2017/6369247)

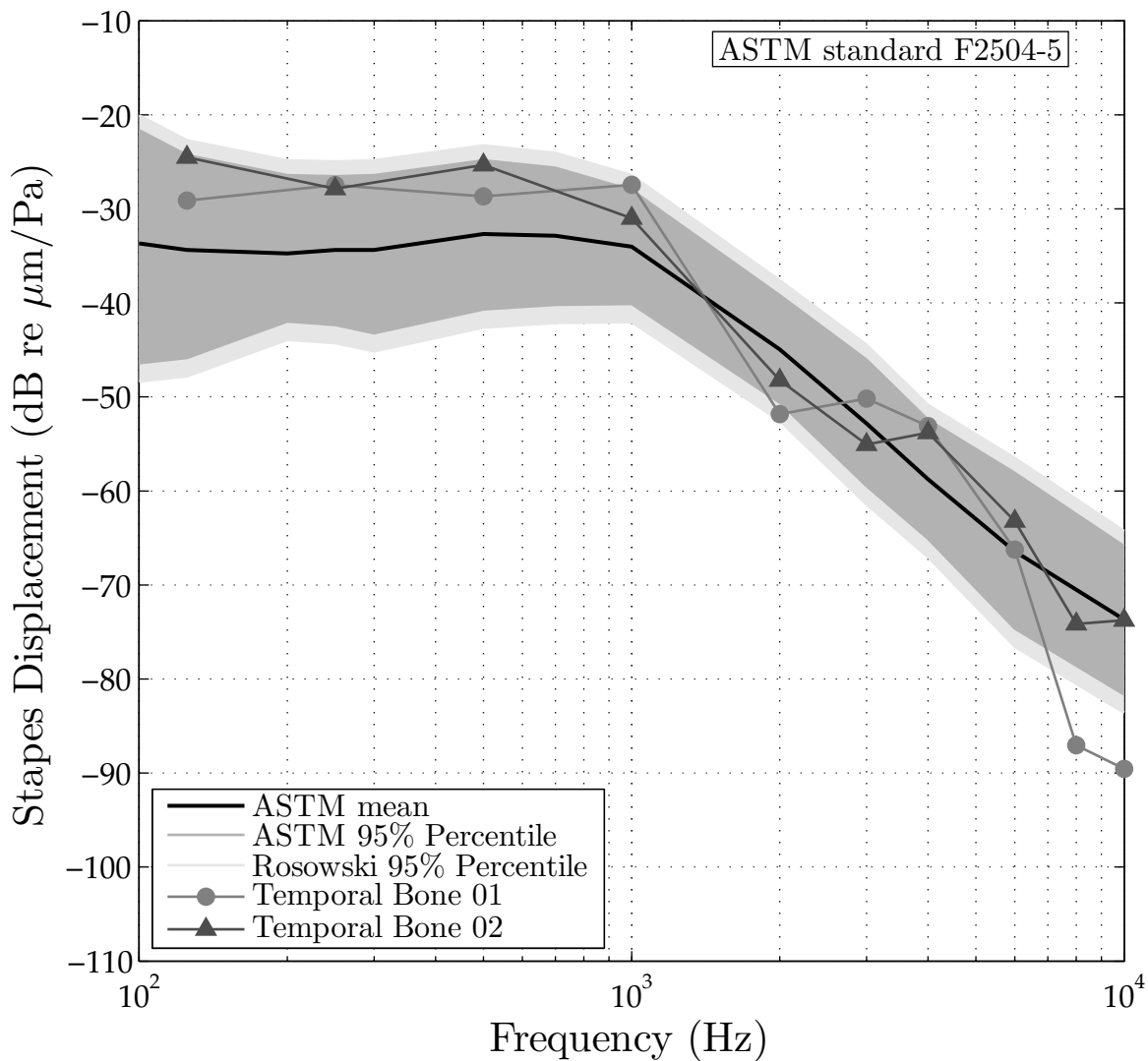

Supplement: Supplementary file 1 — Stapes footplate displacement response to acoustic stimulation of the tympanic membrane (~ 94 dB SPL). The mean responses (ASTM F2504-5) are presented as black line and the 95% percentile range is shown as grey area. The 20% wider range from Rosowski et al. [14] is shown as light grey area. [file 6369247.f1.pdf]
